# Genome-wide association study for leaf area, rachis length and total dry weight in oil palm (*Eleaeisguineensis*) using genotyping by sequencing

**DOI:** 10.1371/journal.pone.0220626

**Published:** 2019-08-07

**Authors:** B. Kalyana Babu, R. K. Mathur, G. Ravichandran, P. Anitha, M. V. B. Venu

**Affiliations:** ICAR-Indian Institute of Oil Palm Research, Pedavegi, West Godavari District, Andhra Pradesh State, India; Faculty of Agriculture (FoA), Sher-e-Kashmir University of Agricultural Sciences and Technology of Kashmir (SKUAST-K), Wadura Campus, INDIA

## Abstract

The marker-trait association for complex traits using genotyping by sequencing (GBS) method is being widely spread in plants. The study aimed to identify significant single nucleotide polymorphism (SNP) associations for rachis length (RL), leaf area (LA) and total dry weight (TrDW) in oil palm among diverse African germplasm. The Illumina NextSeq platform has been used for SNP genotyping and retained 4031 fully informative SNPs after applying the filter criterion. These 4031 SNPs were used for genome wide association study for the above three traits. The LD decay rates of the African germplasm using GBS data of SNP is observed to be 25 Kb at 0.45 of average pair wise correlation coefficient (r^2^). Association mapping led to the identification of seven significant associations for three traits using MLM approach at a P value of ≤ 0.001. Three associations were identified for total dry weight, two each for leaf area index and rachis length. The qtlLA1 was found to be highly significant at a P value of 7.39E^-05^ (18.4% phenotypic variance) which is located on chromosome 4. Two QTLs (qtlLA2 and qtlRL1) were located on chromosome 1, which explained 11.9% and 12.4% of phenotypic variance respectively. Three QTLs for total dry weight were located on chromosome 2, 14 and 16, all-together explained 40% phenotypic variance. The results showed that the SNP-trait associations identified in the present study could be used in selection of elite oil palm germplasm for higher yields.

## Introduction

Oil palm (*Elaeis guineensis* Jacq.) belongs to the family Arecaceae, which is a major contributor (approximately 40%) of world edible vegetable oil. The genome size of oil palm is 1.8 Gb with haploid chromosome number of 16. The genome sequence of an elite dura palm was published [1] and freely available in the open source. It is a perennial crop having potential oil yield capacity of 10 t/ha, against the current yields which varied between 2 to 6 t/ha [2]. Biotechnological tools like molecular markers facilitates in bridging the gap for genetic improvement in yield, oil quality and other important agro-morphological traits of interest. Leaf area and rachis length are found to be highly correlated with oil palm yield which is proven in other crops as well. Hence, leaf area and rachis length are considered as important traits influencing the yield in oil palm. Several studies using GBS for genome-wide association study (GWAS) were reported in rice [3], date palm [4] and oil palm [5], but no reports is available for leaf area, rachis length and total dry weight in oil palm using African germplasm. Molecular markers like RFLPs, AFLPs [6], EST-SSRs [7] and SSRs [5] were used for identification of QTLs for important traits in oil palm. However, genome-wide association study (GWAS) is often hindered due to lack of high diverse genetic pool and well structured germplasm [8]. The GWAS analysis using SNPs emerged as a powerful tool initially in human population to identify complex diseases and then widely implemented in several crops including rice [9], foxtail millet [10–11], maize [12] and in some tree species [13]. The genotyping by sequencing (GBS) method based on restriction digestion of DNA samples has been widely used for identification of QTLs. In oil palm, there are few reports on GBS for GWAS studies for different traits like oil to bunch, oil to dry mesocarp etc. which were mostly based on linkage mapping populations. Recently, Ithnin et al. [14] performed GWAS for oil yield and vegetative traits where they identified 19 QTLs for 8 traits. However, all the studies were reported on F_1_ populations, which lacks clear segregation pattern which can be improved by using advanced segregating generations or highly diverse natural population for association mapping [15]. Till now, no report is available on GWAS for leaf area, rachis length and total dry weight content using SNPs by association mapping.

In perennial crops, natural populations offer advantages like much higher mapping resolution, greater allele number and broader reference population, and less research time in establishing an association [16]. The African sub-continent holds a large diverse germplasm which has highly potential oil yielding and quality traits [17]. In the last 50 years, there was high increase in crude palm oil yield (2.0 to 4.1 t/ha) by conventional breeding, which is still much lower than the theoretical estimates of 18 t/ha [18]. Oil palm yield is highly influenced by leaf area, rachis length and total dry weight content [19]. The shape, leaf area and rachis length of crowns determine light interception and thus influence yield of oil palms (*Elaeis guineensis* Jacq.) by increasing the photosynthetic efficiency as evidenced by high significant correlation observed by several workers [14]. The long breeding cycle of oil palm makes a complicated process for oil palm improvement by conventional breeding. Hence, molecular markers like SNPs plays important role in identification of markers linked to important traits. Hence, in the present study African germplasm were used for identifying SNP-trait associations using GWAS by GBS method. The objectives of the study are 1) Phenotypic characterization of oil palm germplasm for leaf area, rachis length and total dry matter weight, and 2) GWAS analysis for identification of SNP-trait associations for leaf area, rachis length and total dry weight content in oil palm germplasm.

## Methodology

### Plant materials and DNA extraction

Oil palm germplasm representing four African countries were used for SNP genotyping (S1 Fig). The germplasm used in the study were obtained from Malaysian Palm Oil Board (MPOB), Malaysia as part of MoU between ICAR-Indian Institute of Oil Palm Research (ICAR-IIOPR) and MPOB. The palms are grown at the ICAR-Indian Institute of Oil Palm Research (ICAR-IIOPR), Pedavegi, Andhra Pradesh, India experimental fields as per the standard procedures (latitude 16^0^ 48^0^N, longitude 81^0^7^0^E). All the germplasm belongs Dura fruit type. The 96 oil palm germplasm were treated identically in terms of fertilizer application and pest controls. The samples selection was based on the phenotypic variation of selected morphological traits. The experiments were conducted with the consent of Director, ICAR-IIOPR, India. The genomic DNA of oil palm genotypes was isolated by standard method as described by Murray and Thomson [20] with few modifications as described in Babu et al. [21]. The genomic DNA quality and quantity was checked on 0.8% agarose gel using lambda DNA as standard.

### Phenotypic data analysis

**Fig 1:**
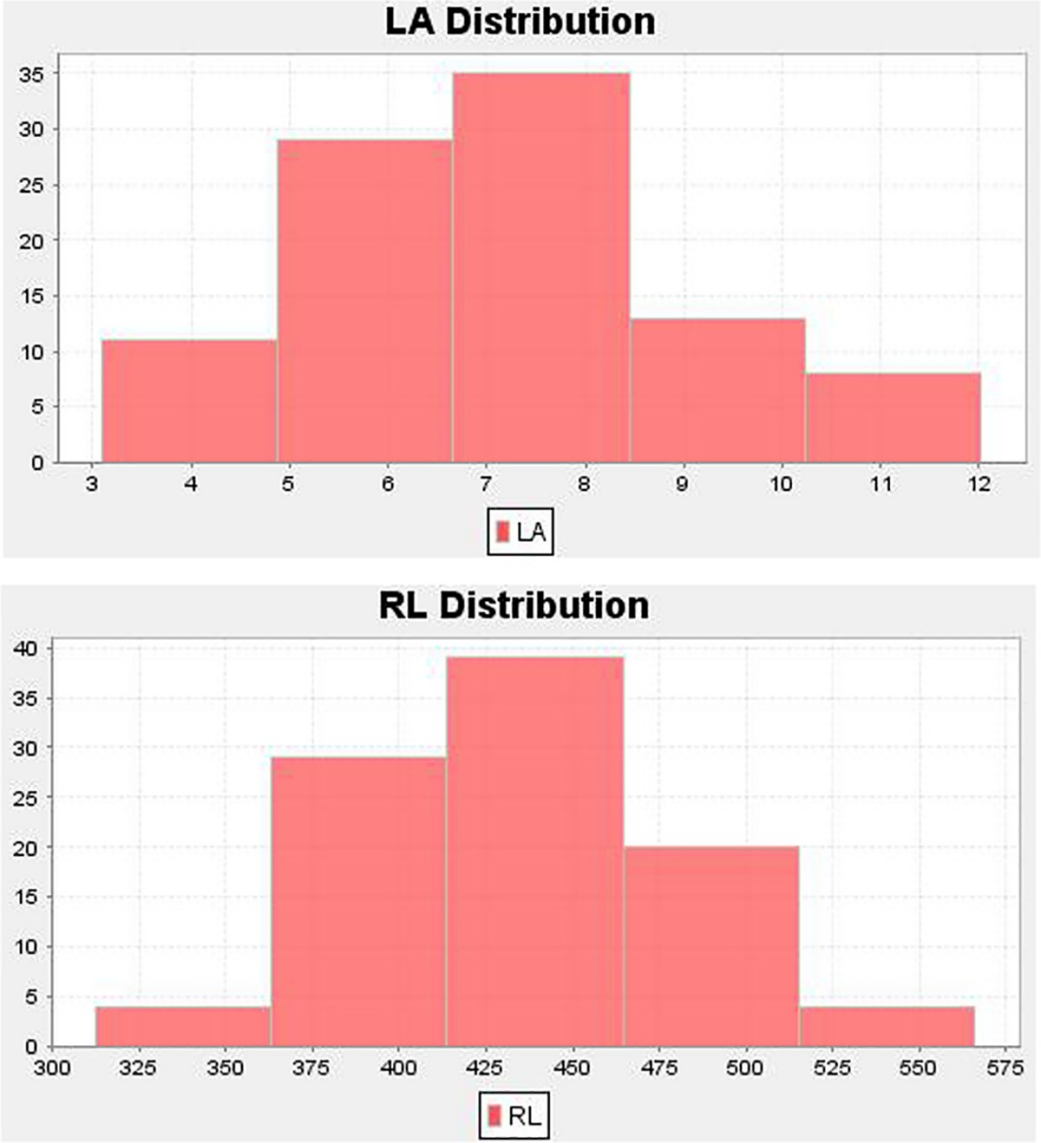
The bar charts for rachis length (RL) and leaf area (LA) of oil palm.

The phenotypic data of leaf area, rachis length and total dry weight content of oil palm were recorded for four consecutive years (2010–2014) according to Corley et al. [22]. The traits used for association mapping are leaf area, rachis length and total dry weight content. Statistical analysis of the traits was estimated for their mean, range, standard deviation and 95% confidence interval. The phenotypic data of rachis length, leaf area and total dry weight is given in S1 Table. The correlation coefficient between the traits and the above statistical parameters calculated using the software JMP [23] and correlation thresholds were considered significant at alpha of 0.001 and 0.01. The distribution of phenotypic data of leaf area and rachis length is given in Fig 1.

### Genotyping by sequencing, SNP calling and annotation

SNP genotyping among the oil palm genotypes was done by following the GBS approach as given by Elshire et al. [24] with ApeKI restriction enzyme used for complexity reduction. The library was prepared using paired end method in the Illumina NextSeq 500 platform. The GBS was done by outsourcing to the Bio Serve (India, Pvt. Ltd). Data normalization has been done at minimum allele frequency (MAF) of 0.05, and by removing 80% missing SNP calls. Only SNP calls >90% has been considered for further analysis. The sequences were mapped to oil palm reference genome [1] and SNPs were called using TASSEL ver. 4.3.10 GBS pipe line [25].

### Genome-wide association mapping

The population structure analysis was done by model based clustering method [26] as adopted in the STRUCTURE v2.3.4 software [27]. Marker-trait associations were performed by using data of 96 oil palm germplasm, SNP genotypic data by GBS approach and Q matrix obtained from the population structure data by using the software TASSEL [28]. The GWAS was performed using MLM approach embedded in the TASSEL software. The kinship matrix was used in addition to the genotypic, phenotypic and Q matrix data in the MLM approach. The significant threshold for the association was set at P of 1X10^-4^. The genome wide patterns of LD decay were estimated by average of R^2^ in distance intervals across the 16 chromosomes of whole genome of oil palm.

## Results and discussion

### Genotyping using GBS for SNP discovery

**Fig 2:**
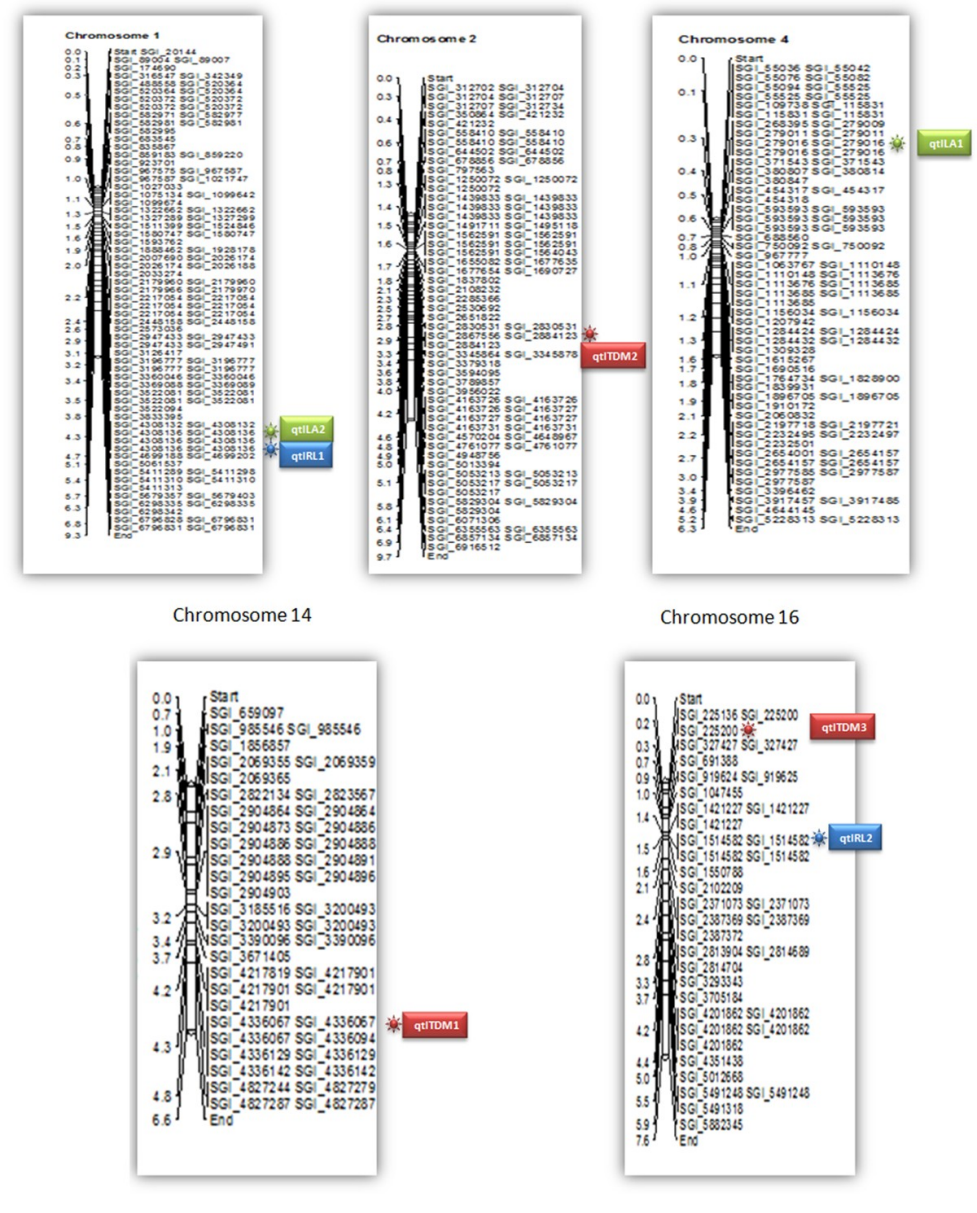
The location of QTLs on oil palm chromosomes.

A set of 96 diverse germplasm from four African countries *viz*., Cameroon, Tanzania, Guinea-Bissau, and Zambia were used for SNP discovery by genotyping by sequencing method. The genomic DNA of 96 African germplasm were subjected to ApeKI digested paired end libraries and sequenced on Illumina NextSeq500 platform. The sequencing resulted in 325 million reads covering 50.78 Gb, at a mean of 3.4 million reads per sample. The sequence data obtained by GBS is lesser than earlier studies [29–31]. Pootakham et al. [13] obtained 524 million reads in F_1_ oil palm population. Teh et al. [32] obtained 900 million raw reads from 132 oil palm F_1_ progeny. The GBS raw data was imputed to SNP calling and data normalization has been done using MAF of 0.05 and by removing 80% missing SNP calls. Only SNP calls >90% has been considered for further analysis. A total of 325 million raw reads generated, of which 2.4 million SNPs passed bar coding and quality thresholds. Finally, 4031SNPs were used for mapping to the reference genome with a range between 157 on chromosome 15 to 455 on chromosome 1 (S2 Table). The physical location of SNPs mapped on the oil palm genome was given in Fig 2. The number of SNPs was similar to the earlier reports [14] where they identified 4451 SNPs across the 422 samples. The SNP allele data files were given in supplementary information (S1 Text).

### The population structure analysis

**Fig 3:**
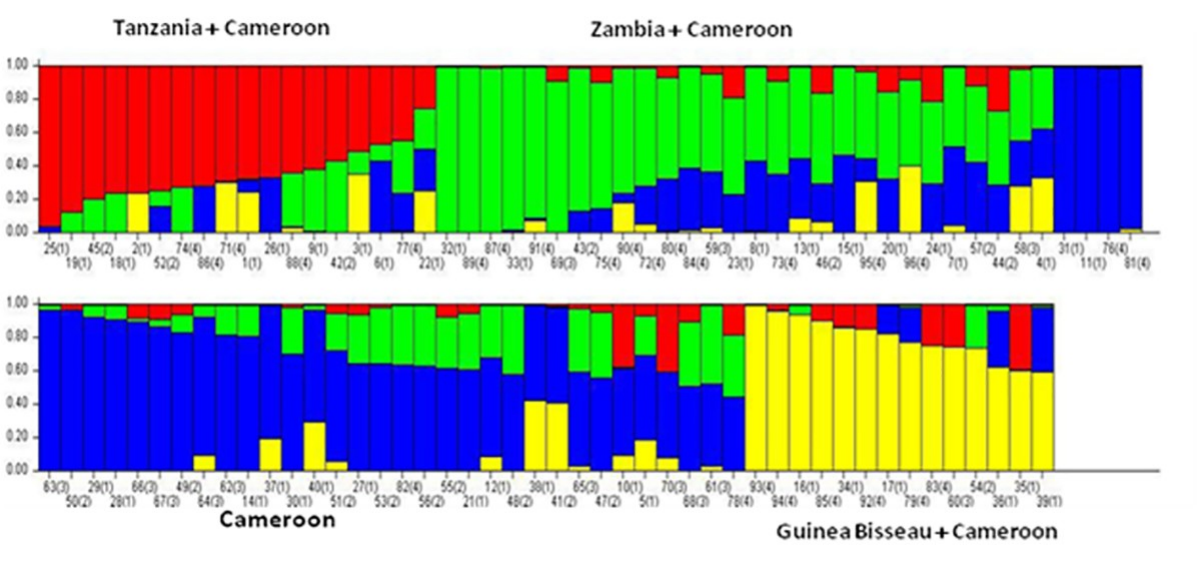
The population structure among the 96 African germplasm as revealed by SNPs by genotyping by sequencing method.

The population structure analysis is conducted among African germplasm of oil palm to estimate the population structure and admixture using SNP markers. To estimate the exact population structure among the oil palm germplasm, Ks were ran from 1 to 10 with 10 iterations and grouping was done based on LnP(D) values. The highest peak value of ΔK was observed at K = 4. High amount of admixture also presented among different groups which may be due to involvement of exotic germplasm. The same type of admixture was also found in earlier reports [32]. The structure analysis revealed that there are four structural patterns existed among the African germplasm which divided them into four groups (Fig 3). The grouping pattern observed to be according the geographic origin of the germplasm to some extent. High amount of admixture of alleles also evident in the populations, which showed a high germplasm exchange between African countries for better breeding programmes. It is also evident that all the present germplasm of oil palm were derived from two Bogor palms, hence high admixture percentage is present.

### Linkage disequilibrium (LD)

The pattern of LD decay is crucial for identifying significant QTLs of any association mapping studies [33] and for interpretation of association peaks [34]. To estimate the mapping resolution for genome wide association mapping, the average extent of LD decay was quantified. The LD decay rates of the oil palm germplasm using GBS data of SNP was 25 Kb at 0.45 of average pair wise correlation coefficient (r^2^). The long range LD in the highly diverse populations can be further explained by breeding selections. The process of selective sweep will much enhance the breeding value of the crops like oil palm. The LD of oil palm plantations in general decays considerably faster than in annual crops like rice and foxtail millet. In both the crops LD decays 100 Kb to 200 Kb (2–5), which decays to 50% of its initial value by 1 Kb with in 150 Kb. This difference may be due to low genome coverage of markers and fewer genotypes in the study. Similar LD decay also observed by Teh et al. [32] where they also found 25Kb and 20Kb at 0.12 and 0.15 of average R^2^ value.

### Genome wide association study (GWAS)

**Fig 4:**
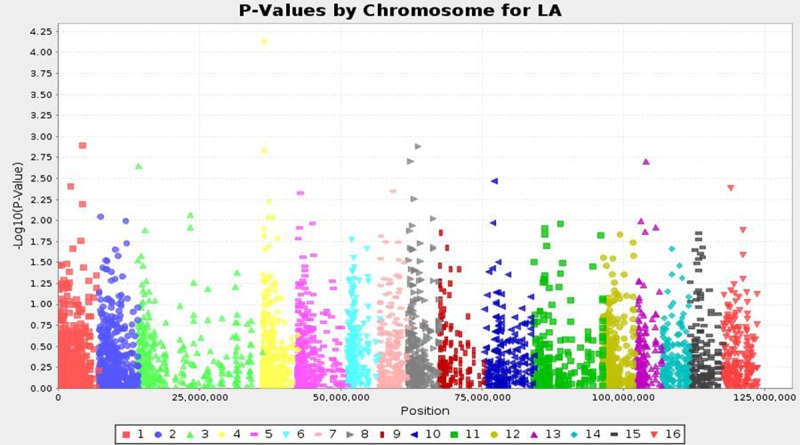
The Man-Hatton plot for leaf area of oil palm as revealed by association mapping.

High yielding oil palm germplasm is an immediate output required for the farming community. There is a big gap in the theoretical CPO yield (18 tons/ha) and global actual average yield (2.0 to 4.0 tons/ha) over the past 50 years [18]. Due to long breeding cycle of oil palm, it became a complicated process in selection of high yielding germplasm. Oil palm yield is highly influenced by leaf area and rachis length. These are complex, quantitative and often controlled by large number of genetic loci [35]. It is important to search for effective QTLs associated with these important traits and utilizing in identification of desirable palms with good yield related traits. Hence, the present investigation aimed to identify significant QTLs using SNPs by genotyping by sequencing method. The study represents the first GWAS study of African germplasm by association mapping approach. Association of leaf area, rachis length and total dry weight using SNPs, resulted identification of seven highly significant QTLs at a P value of ≤ 0.001 by MLM approach. A high significant cut off was given in order to remove the errors generated due to false associations as expected. Three SNP loci were identified to be associated with total dry weight content, followed by two each for leaf area and rachis length. The physical location of QTLs linked to three traits on chromosomes given in Fig 2. All the seven significant QTLs were identified on five chromosomes *viz*., 1, 2, 4, 14, and 16 which explained a phenotypic variance from 11.5 to 18.5%. Two significant QTLs were identified for leaf area, which together explained 30.5% of phenotypic variance. Among them, qtlLA1 (SGI|741241788|) found significant at P value of 7.39E^-05^ (18.5% phenotypic variance). The qtlLA2 (SGI|741241880|) linked at P of 0.001 and explained 12% R^2^ value. These two QTLs located on chromosome 4 and 1 respectively. The manhatton plot for leaf area given in Fig 4.

**Fig 5:**
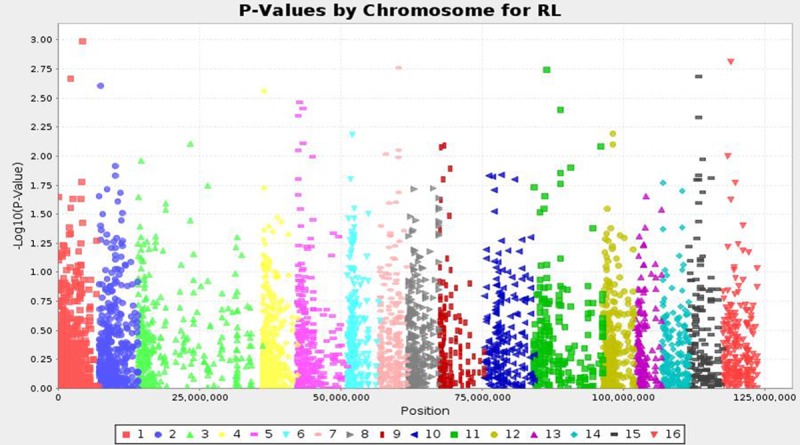
The Man-Hatton plot for rachis length of oil palm.

Three QTLs for total dry weight content and two for rachis length were identified. The loci for total dry weight content were located on chromosome 2, 14 and 16. The QTL (qtlTDM1) located on chromosome 14 found significant at P of 0.0005 which explained 15% phenotypic variance. The three QTLs for total dry weight content together explained 42% of total phenotypic variance. Likewise two QTLs were found significant for rachis length. The manhatton plots for rachis length is given in Fig 5. These two QTLs located on chromosome 1 and 16, together explained 24% of R^2^ value (Table 1). The present study results are similar to the earlier reports [14–15, 31]. Ithnin et al [14] and Billottee et al [31] found significant SNP loci for rachis length on chromosome 2, 4, 10 and 16 on F_1_ progeny by linkage mapping studies. In the present study also QTLs for rachis length identified on 16^th^ chromosome along with unique loci on chromosome 1. The results showed that chromosome 16 harboring the major QTL for rachis length which need attention in cloning the whole gene for rachis length. Billottee et al [31] also identified QTLs for rachis length on 2, 4 and 16 chromosomes. These two reports were based on F_1_ progeny, however the present study is based on genome-wide association study which gives more polymorphism and high precision in identifying QTLs for specific traits. In addition to the above results, the study also identified unique loci for leaf area and rachis length. The identified QTLs in the present study helps in selection of desired oil palm germplasm for high yield characters in different genetic backgrounds which helps in reducing time and increases accuracy.

**Table 1:** The details of significant QTLs identified by GBS method and their P values and phenotypic variances.

Trait	QTL name	Linked SNP loci	P value	R^2^ (%)	Chromosome number
Leaf Area	qtlLA1	SGI|741241788|	7.39E-05	18.5	4
	qtlLA2	SGI|741241880|	0.001	12	1
Total dry weight content	qtlTDM1	SGI|741241610|	0.0005	15	14
	qtlTDM2	SGI|741241857|	0.0009	13.5	2
	qtlTDM3	SGI|741241587|	0.0009	13.5	16
Rachis length	qtlRL1	SGI|741241880|	0.001	12.5	1
	qtlRL2	SGI|741241589|	0.001	11.5	16

## Conclusion

The genotyping by sequencing method used for GWAS study of African germplasm using GBS method to identify the QTLs for three yield related traits *viz*., leaf area, rachis length and total dry weight content. Association mapping resulted in identification of seven highly significant QTLs for different traits at a P value of ≤ 0.001 by MLM approach. More genetic loci were identified to be associated with total dry weight, followed by leaf area and rachis length. The QTLs identified in the present study could be used in selection of elite oil palm germplasm for higher yields.

## Supporting information

S1 Fig The map showing the distribution of oil palm germplasm in four African countries. (DOCX)

S1 Table The phenotypic data of the traits under association mapping. (DOCX)

S2 Table The number of SNPs chromosome wise along with their positions. (DOCX)

S1 Text Allele calling data files for some markers. (DOCX)

## References

[1] Jin J, Lee M, Bai B, Sun Y, Qu J, Rahmadsyah, et al Draft genome sequence of an elite Dura palm and whole-genome patterns of DNA variation in oil palm. DNA Res. 2016; 23(6): 527–533. 10.1093/dnares/dsw036 27426468 PMC5144676

[2] Murphy DJ. The future of oil palm as a major global crop: opportunities and challenges. J Oil Palm Res.2014;26: 1–24.

[3] Fukuoka S, and Okuno K. QTL analysis and mapping of pi21, a recessive gene for field resistance to rice blast in Japanese upland rice. Theor Appl Genet.2001;103:185–190

[4] Mathew LS, Spannagl M. Al-Malki A., George B, Torres MF, Al-Dous EK, Al-Azwani EK, et al First genetic map of date palm (Phoenix dactylifera) reveals long-range genome structure conservation in the palms, BMC Genom. 2014;15: 285. 10.1186/1471-2164-15-285 PMC4023600 24735434

[5] Ting NC, Jansen J, Mayes S, Massawe F, Sambanthamurthi R, Ooi LC, et al High density SNP and SSR-based genetic maps of two independent oil palm hybrids. BMC Genom.2014;15:309 10.1186/1471-2164-15-309 24767304 PMC4234488

[6] Singh R, Tan SG, Panandam JM, Rahman RA, Ooi LC, Low ET, et al Mapping quantitative trait loci (QTLs) for fatty acid composition in an inter specific cross of oil palm. BMCPlant Biol. 2009;9:114 10.1186/1471-2229-9-114 PMC2757029 19706196

[7] Ukoskit K, Vipavee C, Ganlayarat B, Kwanjai P, Sithichoke T, Somvong T. Oil palm (ElaeisguineensisJacq.) linkage map, and quantitative trait locus analysis for sex ratio and related traits Mol Breed. 2014; 33:415–424

[8] Bai B, Le Wang, May Lee, Yingjun Zhang, Rahmadsyah, Yuzer Alfiko, et al Genome-wide identification of markers for selecting higher oil content in oil palm. BMC Plant Biol.2017; 17:93 28558657 10.1186/s12870-017-1045-z PMC5450198

[9] Begum H, Spindel JE, Lalusin A, Borromeo T, Gregorio G, Hernandez J, et al Genome-Wide Association Mapping for Yield and Other Agronomic Traits in an Elite Breeding Population of Tropical Rice (*Oryza sativa*). PLoS ONE. 2015;10(3): e0119873 10.1371/journal.pone.0119873 25785447 PMC4364887

[10] Jia G, Huang X, Zhi H, Zhao Y, Zhao Q, Li W, Chai Y, et al A haplotype map of genomic variations and genome-wide association studies of agronomic traits in foxtail millet (*Setariaitalica*). Nat Genet.2013;45:957–961 10.1038/ng.2673 23793027

[11] Mishra AK, Muthamilarasan M, Khan Y, Parida SK, Prasad M. Genome-wide investigation and expression analyses of WD40 protein family in the model plant foxtail millet (*Setariaitalica* L.). PLoS ONE.2013;9:e86852 10.1371/journal.pone.0086852 PMC3900672 24466268

[12] Ganal MW, Durstewitz G, Polley A, Bérard A, Buckler ES, Charcosset A, et al A Large Maize (Zea mays L.) SNP Genotyping Array: Development and Germplasm Genotyping, and Genetic Mapping to Compare with the B73 Reference Genome. PLoS ONE. 2011; 6(12): e28334 10.1371/journal.pone.0028334 22174790 PMC3234264

[13] Pootakham W, Jomchai N, Areerate P, Jeremy R. Shearman, Sonthirod C, Sangsrakru D, et al Genome-wide SNP discovery and identification of QTL associated with agronomic traits in oil pal, using genotyping–by- sequencing(GBS). BMC Genom.2015;105:288–295. 10.1016/j.ygeno.2015.02.002 25702931

[14] Ithnin M, Xu Y, Marhalil M, Norhalida M, Amiruddin MD, Leslie Low, et al Multiple locus genome-wide association studies for important economic traits of oil palm.Tree Genet Genom. 2017; 13: 103.

[15] Chuenpoma N, Volkaerta H.Association Mapping Identifies Markers Linked with Yield Traits in an Oil Palm Breeding Population.Thai Sci Tech.2016;6(4): 2560.

[16] Mott R, Christopher J. Talbot, Turri MG, Collins AC, Flint J. A method for fine mapping quantitative trait loci in outbred animal stocks. Proc Natl Acad Sci USA.2000;97: 12649–12654 10.1073/pnas.230304397 11050180 PMC18818

[17] Kushairi A, Mohd Din A, Rajanaidu N. Oil palm breeding and seed production. In: MohdBasri W, Choo YM, Chan KW (Eds) Further advances in oil palm research (2000–2010).Malaysian Palm Oil Board, Bangi, Selangor.2011; vol 1: pp 47–101

[18] Corley RHV, Tinker PB. The oil palm 5th ed UK: Wiley; 2015; p. 1–26.

[19] Okoye M, Okwuagwu C, Uguru M, Ataga C, Okolo E. Genotype by trait relations of oil yield in oil palm (ElaeisguineensisJacq.) based on GT biplot. In: 8th African crop science Conference proceedings. 2007; vol. 6: p. 723–8.

[20] Murray MG, Thompson WF. Rapid isolation of high molecular weight plant DNA. Nucleic Acids Res.1980;8:4321–4326 10.1093/nar/8.19.4321 . 7433111 PMC324241

[21] Babu BK, Mathur RK, Kumar PN, Ramajayam D, Ravichandran G, Venu MVB, et al Development, identification and validation of CAPS marker for SHELL trait which governs dura, pisifera and tenera fruit forms in oil palm (*Elaeisguineensis*Jacq.). PLoS ONE.2017;12(2): e0171933 10.1371/journal.pone.0171933 28192462 PMC5305241

[22] Corley RHV, Hardon JJ, Tan GY. Analysis of growth of the oil palm (ElaeisguineensisJacq.) I. Estimation of growth parameters and application in breeding. Euphytica.1971;20:307–315

[23] JMP. 2009; Version 8.02. SAS Institute, Cary, NC

[24] Elshire RJ, Glaubitz JC, Sun Q, Poland JA, Kawamoto K, Buckler ES, et al A robust, simple genotyping-by-sequencing (GBS) approach for high diversity species. PLoS ONE. 2011; 6:E19379 10.1371/journal.pone.0019379 21573248 PMC3087801

[25] Glaubitz JC, Cassteven TM, Lu F, Harriman J, Elshire RJ, Sun Q, et al TASSEL–GBS: a high-capacity genotyping-by-sequencing analysis pipeline. PLoSONE. 2014; 9: e90346 10.1371/journal.pone.0090346 PMC3938676 24587335

[26] Zheng X, Levine D, Shen J, Gogarten SM, Laurie C, Weir BS. A high-performance computing toolset for relatedness and principal component analysis of SNP data. Bioinformatics. 2012; 28:3326–3328. 10.1093/bioinformatics/bts606 23060615 PMC3519454

[27] Pritchard JK, Wen W. Documentation for the structure software, version 2 Department of Human Genetics, University of Chicago, Chicago 2003;http://pritch.bsd.uchicago.edu/software

[28] Bradbury P, Zhang Z, Kroon D, Casstevens T, Ramdoss Y, Buckler ES. TASSEL: software for association mapping of complex traits in diverse samples. Bioinformatics.2007;23(19): 2633–2635 10.1093/bioinformatics/btm308 17586829

[29] Jeennor S, Volkaert H. Mapping of quantitative trait loci (QTLs) for oil yield using SSRs and gene-based markers in African oil palm (ElaeisguineensisJacq.). Tree Genet Genom.2014; 10:1–14

[30] Rance KA, Mayes S, Price Z, Jack PL, Corley RHV. Quantitative trait loci for yield components in oil palm (Elaeisguineensis Jacq). Theor Appl Genet.2001; 103:1302–1310

[31] Billotte N, Jourjon MF, Marseillac N, Berger A, Flori A, Asmady H, et al QTL detection by multi-parent linkage mapping in oil palm (ElaeisguineensisJacq.) Theor Appl Genet.2010;120:1673–1687 10.1007/s00122-010-1284-y 20182696 PMC2859221

[32] Teh CK, Ong AL, Kwong, Apparow S, Chew FT, Mayes S, et al Genome-wide association study identifies three key loci for high mesocarp oil content in perennial crop oil palm. Scientific Reports.2016;6: 19075, 10.1038/srep19075 26743827 PMC4705476

[33] Kim S, Plagnol V, Hu TT, Christopher T, Richard MC, Stephan O, et al Recombination and linkage disequilibrium in Arabidopsis thaliana. Nat Genet.2007;39(9):1151–1155. 10.1038/ng2115 17676040

[34] Huang X, Wei X, Sang T, Zhao Q, Feng Q, Zhao Y, et al Genome-wide association studies of 14 agronomic traits in rice landraces. Nat Genet. 2010;42(11):961–967. 10.1038/ng.695 20972439

[35] Kainer D, Lanfear R, Foley WJ, Külheim C. Genomic approaches to selection in outcrossing perennials: focus on essential oil crops. Theor Appl Genet. 2015; 128 (12): 2351–65. 10.1007/s00122-015-2591-0 26239409

