# Supplementary material for: Genome-wide association study for leaf area, rachis length and total dry weight in oil palm (Eleaeisguineensis) using genotyping by sequencing
Source: PLoS One. 2019 Aug 7;14(8):e0220626. doi: 10.1371/journal.pone.0220626 (PMC6685610; doi:10.1371/journal.pone.0220626)
Supplement: S1 Fig — (DOCX) [file pone.0220626.s001.docx]

S1 Fig
