# Supplementary material for: Genome-wide association study for leaf area, rachis length and total dry weight in oil palm (Eleaeisguineensis) using genotyping by sequencing
Source: PLoS One. 2019 Aug 7;14(8):e0220626. doi: 10.1371/journal.pone.0220626 (PMC6685610; doi:10.1371/journal.pone.0220626)
Supplement: S1 Table — (DOCX) [file pone.0220626.s002.docx]

S1 Table

| Palm No | RL (Rachis length) (cm) | LA (Leaf Area) (sq.cm) | TrDW (Total dry weight) (Kg) |
| --- | --- | --- | --- |
| 27 | 423.00 | 5.46 | 87.23 |
| 28 | 431.00 | 6.62 | 154.86 |
| 31 | 488.67 | 9.43 | 47.02 |
| 42 | 450.67 | 10.02 | 36.67 |
| 44 | 426.33 | 5.99 | 40.67 |
| 45 | 423.33 | 5.83 | 48.52 |
| 46 | 413.50 | 4.45 | 56.95 |
| 56 | 427.00 | 6.85 | 36.06 |
| 60 | 474.50 | 9.31 | 33.56 |
| 62 | 498.33 | 10.90 | 30.75 |
| 66 | 511.50 | 7.82 | 28.35 |
| 72 | 435.67 | 4.87 | 90.25 |
| 73 | 395.00 | 8.46 | 96.76 |
| 74 | 374.00 | 4.27 | 93.87 |
| 75 | 496.67 | 7.97 | 110.91 |
| 77 | 488.00 | 5.77 | 30.65 |
| 82 | 422.33 | 7.34 | 134.76 |
| 83 | 419.33 | 10.46 | 31.57 |
| 92 | 446.33 | 10.12 | 59.04 |
| 99 | 500.00 | 10.76 | 52.24 |
| 105 | 410.33 | 6.82 | 33.23 |
| 119 | 402.33 | 4.95 | 43.44 |
| 126 | 412.33 | 6.39 | 107.75 |
| 127 | 417.00 | 5.86 | 85.91 |
| 129 | 468.67 | 8.91 | 89.81 |
| 130 | 320.45 | 5.32 | 80.15 |
| 133 | 484.00 | 8.28 | 112.77 |
| 147 | 485.67 | 8.38 | 47.58 |
| 174 | 340.33 | 5.99 | 52.58 |
| 180 | 439.67 | 7.47 | 178.30 |
| 181 | 458.67 | 6.00 | 112.35 |
| 185 | 325.50 | 3.09 | 34.24 |
| 187 | 407.67 | 6.13 | 105.96 |
| 188 | 432.33 | 6.75 | 95.29 |
| 197 | 475.67 | 8.63 | 43.09 |
| 198 | 373.00 | 7.36 | 36.50 |
| 199 | 370.00 | 4.53 | 32.52 |
| 202 | 379.00 | 3.90 | 43.54 |
| 203 | 467.00 | 8.07 | 32.47 |
| 207 | 490.00 | 8.35 | 40.41 |
| 213 | 458.67 | 7.37 | 31.02 |
| 235 | 425.00 | 7.73 | 174.12 |
| 236 | 402.00 | 7.27 | 123.99 |
| 242 | 495.33 | 9.92 | 185.77 |
| 243 | 456.67 | 7.82 | 141.36 |
| 254 | 458.67 | 7.64 | 31.06 |
| 287 | 398.00 | 5.16 | 37.85 |
| 294 | 441.00 | 7.48 | 39.17 |
| 298 | 422.67 | 7.80 | 189.38 |
| 300 | 431.67 | 6.88 | 191.26 |
| 301 | 456.00 | 8.68 | 196.62 |
| 318 | 461.33 | 8.39 | 34.75 |
| 336 | 312.33 | 3.82 | 29.26 |
| 345 | 428.00 | 6.77 | 154.46 |
| 346 | 409.33 | 5.93 | 129.47 |
| 375 | 466.33 | 8.45 | 41.77 |
| 376 | 505.67 | 9.21 | 43.02 |
| 385 | 418.00 | 6.20 | 41.68 |
| 396 | 468.00 | 7.92 | 36.76 |
| 406 | 395.33 | 6.53 | 32.81 |
| 407 | 368.33 | 4.67 | 34.78 |
| 409 | 396.33 | 5.71 | 41.53 |
| 410 | 516.33 | 7.68 | 33.74 |
| 412 | 406.67 | 7.02 | 45.96 |
| 415 | 382.67 | 5.05 | 44.67 |
| 416 | 464.00 | 8.47 | 50.04 |
| 417 | 364.33 | 4.47 | 40.25 |
| 419 | 333.00 | 6.10 | 42.19 |
| 423 | 450.67 | 7.26 | 47.34 |
| 425 | 409.33 | 5.58 | 52.99 |
| 427 | 418.33 | 6.98 | 55.49 |
| 429 | 429.00 | 7.67 | 24.64 |
| 434 | 409.67 | 6.19 | 46.30 |
| 436 | 446.67 | 7.31 | 58.19 |
| 437 | 455.00 | 5.50 | 52.96 |
| 438 | 430.00 | 6.19 | 38.60 |
| 443 | 420.50 | 5.64 | 76.76 |
| 448 | 381.00 | 5.62 | 36.80 |
| 449 | 371.33 | 4.51 | 52.18 |
| 452 | 450.00 | 7.73 | 34.98 |
| 453 | 369.00 | 4.70 | 48.02 |
| 454 | 502.67 | 10.55 | 61.58 |
| 472 | 379.00 | 6.45 | 58.66 |
| 473 | 393.33 | 5.48 | 62.82 |
| 478 | 378.00 | 5.82 | 44.56 |
| 479 | 484.67 | 9.16 | 45.86 |
| 480 | 429.67 | 6.83 | 51.77 |
| 482 | 440.33 | 5.97 | 27.03 |
| 484 | 431.33 | 8.38 | 35.05 |
| 485 | 369.67 | 6.75 | 41.51 |
| 486 | 409.33 | 6.65 | 54.12 |
| 534 | 565.67 | 11.19 | 50.33 |
| 535 | 451.67 | 7.49 | 33.23 |
| 539 | 510.33 | 11.40 | 46.20 |
| 542 | 566.00 | 12.02 | 36.50 |
| 543 | 460.67 | 8.07 | 48.66 |
| Mean | 431.87 | 7.12 | 65.11 |
| SD | 25.57 | 1.85 | 23.56 |
