# Supplementary material for: Genome-wide association study for leaf area, rachis length and total dry weight in oil palm (Eleaeisguineensis) using genotyping by sequencing
Source: PLoS One. 2019 Aug 7;14(8):e0220626. doi: 10.1371/journal.pone.0220626 (PMC6685610; doi:10.1371/journal.pone.0220626)
Supplement: S2 Table — (DOCX) [file pone.0220626.s003.docx]

S2 Table

| Chromosome | No. of SNP loci | Heterozygote | Start | End |
| --- | --- | --- | --- | --- |
| 1 | 455 | 0.371 | 20142 | 7225433 |
| 2 | 371 | 0.341 | 2860 | 6916531 |
| 3 | 321 | 0.358 | 43766 | 22063540 |
| 4 | 358 | 0.385 | 2708 | 6196434 |
| 5 | 275 | 0.358 | 2456 | 8791756 |
| 6 | 261 | 0.371 | 25814 | 5803614 |
| 7 | 240 | 0.412 | 191235 | 5192687 |
| 8 | 265 | 0.393 | 11108 | 5519905 |
| 9 | 159 | 0.376 | 60060 | 7952377 |
| 10 | 201 | 0.398 | 13184 | 8825285 |
| 11 | 198 | 0.342 | 1246 | 12212450 |
| 12 | 240 | 0.400 | 98045 | 5782788 |
| 13 | 148 | 0.385 | 4927 | 4691843 |
| 14 | 188 | 0.392 | 47671 | 4827293 |
| 15 | 157 | 0.388 | 6594 | 5995646 |
| 16 | 194 | 0.381 | 74920 | 6175046 |
| Entire genome | 4031 | 0.380 |  | |
