# Supplementary material for: Genome-wide association study for leaf area, rachis length and total dry weight in oil palm (Eleaeisguineensis) using genotyping by sequencing
Source: PLoS One. 2019 Aug 7;14(8):e0220626. doi: 10.1371/journal.pone.0220626 (PMC6685610; doi:10.1371/journal.pone.0220626)
Supplement: S1 Text — (DOCX) [file pone.0220626.s004.docx]

S1 Text

rs# alleles chrom pos strand assembly# center protLSID assayLSID panelLSID QCcode Zambia_105 Guinea_119 Cameron_126 Cameron_127 Cameron_129 Cameron_130 Cameron_133 Tanzania_147 Guinea_174 Cameron_180 Cameron_181 Cameron_185 Cameron_187 Cameron_188 Cameron_197 Tanzania_198 Guinea_199 Guinea_202 Zambia_203 Zambia_207 Zambia_213 Cameron_235 Cameron_236 Cameron_242 Cameron_243 Zambia_254 Cameron_27 Cameron_28 Guinea_287 Cameron_294 Cameron_298 Cameron_300 Cameron_301 Tanzania_31 Tanzania_318 Guinea_336 Cameron_345 Cameron_346 Cameron_375 Cameron_376 Cameron_385 Cameron_396 Zambia_406 Guinea_407 Guinea_409 Zambia_410 Tanzania_412 Guinea_415 Zambia_416 Guinea_417 Tanzania_419 Zambia_42 Zambia_423 Guinea_425 Tanzania_427 Zambia_429 Cameron_434 Cameron_436 Cameron_437 Zambia_438 Tanzania_443 Zambia_448 Guinea_449 Zambia_44 Zambia_452 Guinea_453 Tanzania_454 Zambia_45 Guinea_46 Guinea_472 Guinea_473 Guinea_478 Tanzania_479 Tanzania_480 Cameron_482 Zambia_484 Zambia_485 Tanzania_486 Cameron_534 Cameron_535 Zambia_539 Zambia_542 Zambia_543 Zambia_56 Zambia_60 Zambia_62 Cameron_66 Cameron_72 Cameron_73 Cameron_74 Cameron_75 Cameron_77 Cameron_82 Zambia_83 Tanzania_92 Zambia_99

SGI|741241878|REF|NW_011550610.1|_20142 A/- 1 20142 + NA NA NA NA NA NA A A A A A A A A A 0 A A A A N A A A A A A A A A A A A A A A A A A A A A N A A A A A A 0 A A 0 A A A A A 0 A 0 A A 0 A A 0 A 0 A 0 A A A A A A A 0 A A A A 0 A A A A 0 A A A A A A A A A A A A A

SGI|741241878|REF|NW_011550610.1|_20143 A/- 1 20143 + NA NA NA NA NA NA A 0 0 0 A 0 0 0 0 0 A 0 A A 0 0 0 0 A 0 A 0 0 0 0 0 0 0 0 0 A A 0 0 0 0 N 0 0 A 0 A A 0 0 0 0 0 0 0 A 0 0 0 0 0 0 0 0 0 0 0 0 A 0 0 0 A 0 0 A 0 0 0 0 0 0 0 0 0 0 0 0 0 A A A A A A 0 A 0 0 A 0

SGI|741241873|REF|NW_011550615.1|_36511 T/G 1 36511 + NA NA NA NA NA NA K K K T K T K K K K K K K T T K K K K T K T K T K T K T T T T T T K T T N K K K T T N T T T T T T T G K T T T T T T T T T T K K T K T K K T T K G T K K T T T K K K K T K K K K K G G K G K T K

SGI|741241875|REF|NW_011550613.1|_52453 -/G 1 52453 + NA NA NA NA NA NA - - - - - - - - 0 - 0 0 0 0 N - 0 - - - 0 - - 0 0 - - 0 - 0 - - - - - 0 N - - - - - N - - - - - - - - - - - - - - - - - - - - - - - - - - - - - - - - - - - - - - - - - - - - - - - 0 - - - 0 -

SGI|741241870|REF|NW_011550618.1|_55251 G/- 1 55251 + NA NA NA NA NA NA G 0 G 0 0 G G G 0 0 0 0 G G G 0 0 0 0 0 0 G 0 0 0 0 0 0 0 0 0 0 G 0 0 G N 0 0 0 0 G N G 0 0 G 0 0 G 0 0 0 G 0 G 0 G 0 0 0 0 0 G 0 0 0 G 0 0 0 0 0 0 0 0 0 0 0 0 0 0 0 0 G 0 0 G G G 0 0 0 G 0 G

SGI|741241870|REF|NW_011550618.1|_67199 A/- 1 67199 + NA NA NA NA NA NA A A A A A A A A A A A A A A A A A A A A A A A A A A A 0 A A A A A 0 A A N A A A A A N A A A A A A A A 0 A A A A A A A A A A A 0 0 A A 0 0 A A A A A A A A A A A A A A 0 0 0 A 0 0 0 0 A A A A A

SGI|741241870|REF|NW_011550618.1|_67203 A/- 1 67203 + NA NA NA NA NA NA A A A A A A A A 0 A A A A A A 0 A 0 A 0 A 0 A 0 A 0 A A A A A A 0 A A A N 0 A 0 A A N 0 A A 0 A A 0 A A A 0 A A A A A 0 0 0 A A 0 0 A A A A A A A A A 0 A A 0 A 0 A 0 A A A 0 A A 0 A A A A A A

SGI|741241870|REF|NW_011550618.1|_67219 A/- 1 67219 + NA NA NA NA NA NA A A A A 0 A A 0 0 A 0 A A A 0 0 A 0 0 A 0 A A A 0 A A A A A A 0 A A 0 0 N A A A A 0 N 0 A A 0 A 0 A A A 0 A 0 A A A A A A 0 A A A A A A A 0 A A A A A A A A A A A A 0 A A A A A A 0 A A A A A 0

SGI|741241871|REF|NW_011550617.1|_77434 A/- 1 77434 + NA NA NA NA NA NA A A A A A A A A A A A A A A A A A A A A A A A A A A A A A A A A A A A A N A A - - - - - - - - - - - - A - - - - - - - - - - - A - - - A A - - - - - - - - - - - - - - A A A A A A A A A A A A A

- - -
